# Arbuscular Mycorrhizal Fungi Improve *Lycium barbarum* Potassium Uptake by Activating the Expression of *LbHAK*

**DOI:** 10.3390/plants13091244

**Published:** 2024-04-30

**Authors:** Yongxin Zhang, Xia Han, Wei Ren, Haoqiang Zhang, Ming Tang

**Affiliations:** 1 College of Forestry, Northwest A&F University, Yangling 712100, China; zyx0213g@163.com (Y.Z.); 2018060255@nwafu.edu.cn (X.H.); weirensx@gmail.com (W.R.); 2 Shaanxi Engineering Research Center of Forage Plants of the Loess Plateau, College of Life Sciences, Yulin University, Yulin 719000, China; 3 State Key Laboratory of Conservation and Utilization of Subtropical Agro-Bioresources, College of Forestry and Landscape Architecture, South China Agricultural University, Guangzhou 510642, China

**Keywords:** arbuscular mycorrhizal, *LbHAK*, potassium, phosphorus, water

## Abstract

Arbuscular mycorrhizal (AM) fungi can establish a mutualistic relationship with the roots of most terrestrial plants to increase plant nutrient uptake. The effects of potassium uptake and transport by AM symbiosis are much less reported compared to other nutrients. In this research, a heterologous yeast system was used to verify that the LbHAK has capacity for potassium uptake. The split-roots system implemented using seedlings of *Lycium barbarum* confirmed that *R. irregularis* locally induced *LbHAK* expression, which means that *LbHAK* is only expressed in mycorrhizal roots. Furthermore, the impacts of overexpression of *LbHAK* on the growth, nutrients and water uptake, and transport of mycorrhizal tobacco (inoculation with *Rhizophagus irregularis*) at 0.2 mM and 2 mM K conditions were assessed. The mycorrhizal tobacco growth and potassium accumulation were significantly enhanced through *LbHAK* overexpression in tobacco. In addition, overexpression of *LbHAK* substantially enhanced phosphorus content, while stimulating the expression of *NtPT4*, *Rir-AQP1*, and *Rir-AQP2* in mycorrhizal tobacco. Moreover, *LbHAK* overexpression greatly promoted AM colonization. *LbHAK* has a potential role in facilitating potassium absorption through the mycorrhizal pathway, and overexpression of *LbHAK* in tobacco may promote the transport of potassium, phosphorus, and water from AM fungi to tobacco. These data imply the important roles played by the *LbHAK* in AM-fungi-induced potassium uptake in *L. barbarum* and in improving plant nutrients and AM colonization.

## 1. Introduction

More than 80% of terrestrial plants can be colonized by arbuscular mycorrhizal (AM) fungi, which belong to monophyletic phylum Glomeromycota [1]. The symbiotic system between AM fungi and plants improves plant growth, particularly in nutrient-limited conditions [2,3]. Mycorrhizal plants can not only directly uptake water and nutrients through plant roots, but also indirectly through AM fungal hyphae, which expands the absorption area of plants and improves the nutrition and water status of plants and is also known as the mycorrhizal pathway [4]. Many studies have provided evidence indicating that the mycorrhizal pathway is crucial for the absorption of phosphate (Pi), and several Pi transporters in plants have been identified, which are induced by AM fungi, such as *MtPT4*, *NtPT4*, and *LbPT4* [5,6,7]. The increasing evidence shows that the mycorrhizal pathway can also uptake substantial amounts of nitrogen, copper, and sulfur [8,9,10,11,12].

Potassium is a vital nutritional element in plants involved in many metabolic processes [13,14]. Potassium plays important roles in plant growth and development, as well as in coping with adverse conditions, especially drought stress [13,15,16,17]. Higher cytosolic potassium/sodium ratios contribute to enhanced salt tolerance in plants [18]. Potassium could promote chlorophyll synthesis and increases the rate of photosynthesis, which enhances plant resistance to drought [19,20]. Furthermore, potassium application can increase the activities of superoxide dismutase (SOD), catalase (CAT), and peroxidase (POD) in plants, thereby promoting the capacities of scavenging reactive oxygen species and reducing the damage caused by the accumulation of oxidative substances by drought stress [17,21,22]. Additionally, potassium also contributes to plant cell osmosis, promoting the accumulation of substances such as proline, helping plants overcome drought environments [23,24]. Application of potassium fertilization in *Zea mays* L. significantly reduces water consumption and increases water use efficiency [25]. In addition to drought stress, potassium fertilization also promotes the uptake and transport of nitrogen and phosphorus, thereby improving the nutritional conditions of plants [26]. However, potassium availability fluctuates widely in soils around the world and is often seen as a constraint on plant growth [27,28]. Thus, improving the capacity of potassium uptake is critical for plant growth and adaptation to stress conditions [13].

Garcia and Zimmermann (2014) [29] reported a strong potassium accumulation in AM fungi and in some plant tissues, implying the possibility of mycorrhizal potassium uptake. Moreover, previous studies discovered the AM-fungi-induced potassium transporters in *Lotus japonicus* (*LjHAK*) and tomato (*SlHAK10*) and proved that *SlHAK10* mediated the mycorrhizal potassium uptake pathway [30,31]. *Lycium barbarum* L. is an important traditional medical plant in northwestern China, where water is one of main limiting factors for plant growth [6,32]. In the arid and semi-arid regions, the mobility of readily available potassium is reduced, making it more difficult to utilize, so plants often face a lack of potassium nutrition under drought conditions [24]. In this study, the expression of a putative orthologue gene of *SlHAK10* (designated as *LbHAK*) in mycorrhizal *L. barbarum* roots was significantly induced compared with non-mycorrhizal roots [33]. We evaluated the capability of *LbHAK* in potassium uptake by conducting complementation analysis in a yeast (*Saccharomyces cerevisiae*) mutant deficient in potassium uptake (CY162). A split-root experiment with *L. barbarum* seedlings was carried out to investigate whether the expression of *LbHAK* was systemically or locally induced by AM fungi. Overexpression of *LbHAK* driven by the CaMV35S promoter in tobacco was conducted to further evaluate the function of *LbHAK* by comparing the growth, AM colonization, nutrient status, and gene expression with wild-type tobacco under different potassium conditions.

## 2. Materials and Methods

### 2.1. Growth Substrate and AM Fungal Inoculum

The growth substrate was a mixture of sand and vermiculite (1:1, *v*:*v*). The sand was sieved through a 2 mm sieve and thoroughly washed with tap water. Sand and vermiculite were autoclaved at 121 °C for 2 h twice with one-day interval.

The inoculum of *Rhizophagus irregularis* (BGC BJ109) was propagated with *Plantago asiatica* for 12 months. The inoculum consisted of the sandy substrate that contained spores, mycelium, and colonized root fragments. The inoculum consisted of the sandy substrate that contained spores, mycelium, and colonized root fragments containing 150 propagules per gram, determined by the most probable number method.

### 2.2. Clone and Bioformation Analysis of LbHAK

The partial sequence of *LbHAK* was obtained by independent de novo transcriptome sequencing (unpublished). The full *LbHAK* sequence was amplified by the 5′ and 3′ rapid amplification of cDNA ends (RACE) procedure using the SMARTer™ RACE cDNA Amplification Kit (Clontech Laboratories, Inc., Mountain View, CA, USA). The primers used are listed in Table S1.

The Open Reading Frame (ORF) Finder (https://www.ncbi.nlm.nih.gov/orffinder/ (accessed on 3 April 2024)) in NCBI was utilized for ORF analysis and amino acid sequence prediction. The ProtParam tool (http://web.expasy.org/protparam/ (accessed on 3 April 2024)) was utilized for the prediction of the molecular weight and isoelectric point of proteins. The subcellular localization was predicted by TargetP 2.0 Server (http://www.cbs.dtu.dk/services/TargetP/ (accessed on 3 April 2024)). DeepTMHMM (https://dtu.biolib.com/DeepTMHMM (accessed on 3 April 2024)) was used to predict transmembrane helices in proteins. The neighbor-joining (NJ) method was employed by MEGA 6.0.6 to construct a phylogenetic tree.

### 2.3. Heterologous Expression of LbHAK in Yeast

The *LbHAK* coding sequence was inserted into the KpnI to SacI sites of pYES2 yeast expression vector. The recombinant vector and pYES2 vector (Table S2) were separately introduced into the yeast potassium-uptake-defective mutant strain CY162 (*MATα ura3-52 his4-15 trk1Δ trk2Δ1*::*pCK64*) (Table S1) [34]. The wild-type yeast strain was BY4741 (Table S2) [35]. The LiAC/single-stranded DNA/polyethylene glycol method was utilized for yeast cell transformation, and the growth was measured on AP medium (supplemented with galactose) as suggested by Horie et al. [36].

### 2.4. Split-Root Experiment Analysis

A split-root experiment was conducted to test whether *LbHAK* was only expressed in AM-fungi-colonized root parts of plants (Figure S1). The preparation of *L. barbarum* seedlings, the split-root system, the setup of mycorrhizal (AM) and non-mycorrhizal (NM) treatments, and the cultivation and harvest were in accordance with the description of Han et al. [12].

### 2.5. Overexpression of LbHAK in Tobacco

The complete coding sequence of *LbHAK* was cloned and integrated to the pROKII vector (Table S2) at the SmaI to SacI sites. The *LbHAK*-*pROKII* recombinant vector was transformed into the *Agrobacterium tumefaciens* GV3101. The leaf disc method was employed to accomplish *A. tumefaciens*-mediated transformation of tobacco (*Nicotiana tabacum*), followed by cultivation of the transformed plants on Murashige and Skoog medium.

Wild-type (WT) and *LbHAK*-overexpression (OE) tobacco lines were used for the pot experiment. T0 seeds of WT and OE tobacco were surface-disinfected with 5% NaClO for 5 min and washed 3 times with sterile water. The sterile seeds were then germinated on moist filter paper at 28 °C in darkness. The germinated seeds were planted in a tray filled with growth substrate same as mentioned above for 5 weeks. Uniform tobacco seedlings of two lines were selected for transplantation. The seedlings were grown in a pot (10 cm × 10 cm × 9.6 cm) containing 400 g of growth substrate. The seedlings in AM treatment received 10 g AM fungal inoculum beneath roots during transplantation, while seedlings in NM treatment received autoclaved inoculum (121 °C for 2 h) and 10 mL filtrate (<20 μm nylon mesh) of inoculum. After transplantation, seedlings of tobacco were watered daily and irrigated weekly with 20 mL Hoagland solution containing 10% phosphate (0.1 mM KH_2_PO_4_) to ensure the colonization of AM fungi. After 3 weeks of growth, two concentrations (0.2 and 2 mmol K kg^−1^ growth substrate) of K_2_SO_4_ solution (20 mL) were applied to the tobacco seedlings, and the growth continued for another 3 weeks. There were 8 treatments in total. Each treatment had four replicates, and 2 seedlings were included in each replicate. The pot experiment was carried out in a controlled greenhouse environment where there was 16 h light per day, along with a temperature range of 24–28 °C and a relative humidity range of 40–60%.

### 2.6. Measurement of Plant Biomass, Mycorrhizal Colonization, and Potassium and Phosphorus Concentrations

At harvest, the fresh weight of the shoot and root of tobacco in the pot experiment were recorded, respectively. Parts of the shoots and roots of tobacco were killed green at 110 °C for 15 min, and then dried at 65 °C until the samples reached a constant weight. The remaining portions of shoots and roots from the split-root experiment and pot experiment were immediately frozen and ground into a powder using liquid nitrogen, and then stored at −80 °C until further use in relative gene expression analyses.

Part of the roots from the split-root experiment and pot experiment were cut into 1 cm fragments and stained with trypan blue [37], and then mycorrhizal colonization rate was assessed using the magnified intersection method with a light microscope [38].

Plant dry materials were ground and digested in accordance with Han et al. [39]. The flame photometer was used to measure potassium concentration and using method of molybdenum yellow colorimetric to determine phosphorus concentration [40]. The content was calculated by the concentration and dry weight of potassium and phosphorus.

### 2.7. Relative Gene Expression Analysis

Total RNA was extracted by the E.Z.N.ATM Plant RNA kit (Omega Bio-Tek, Norcross, GA, USA). Following the instructions provided by the supplier, 1 μg of high-quality RNA was digested DNAse utilizing the Hifair^®^ Ⅲ 1st Strand cDNA Synthesis SuperMix for qPCR (gDNA digester plus) (YEASEN Bio, Shanghai, China). After DNA digestion, the control PCR was applied to check the possible trace DNA contamination. Afterwards, the RNA was transcribed to cDNA using the above-mentioned kit, and the cDNA was then employed as a template for PCR reactions. The transcript accumulation analysis of *LbHAK*, *GintEF1α*, *NtPT4*, *Rir-AQP1*, and *Rir-AQP2* in roots and *R. irregularis* hyphae was performed by SYBR green-based qRT-PCR. The *Lbactin* and *NtEF1a* were used as an internal control of *L. barbarum* and tobacco roots, respectively. The *GintEF1α* was used as an internal control of *R. irregular* hyphae. The specific primers for qRT-PCR can be found in Table S1. The reactions of qRT-PCR were performed by the description of Han et al. [12]. The transcript abundance of *LbHAK* in the overexpression lines of tobacco was also evaluated by qRT-PCR as mentioned above; the *NtEF1a* gene was used as internal reference gene to normalize the expression data. The relative expressions were determined using the equation 2^−ΔΔCT^ [41].

### 2.8. Statistical Analysis

IBM SPSS Statistics 21.0 software (IBM, Armonk, NY, USA) was conducted for statistical analysis. Data were analyzed by a three-way ANOVA (tobacco lines, AM status, and potassium status) and Duncan’s test. Spearman’s correlation was used for correlation analyses with data of root nutrient contents, colonization rate, and gene expression in tobacco roots and *R. irregularis*. Origin Pro 2023 (Origin Lab, Northampton, MA, USA) was utilized to create the figures.

## 3. Results

### 3.1. Identification and Functional Analysis of LbHAK

**Figure 1:**
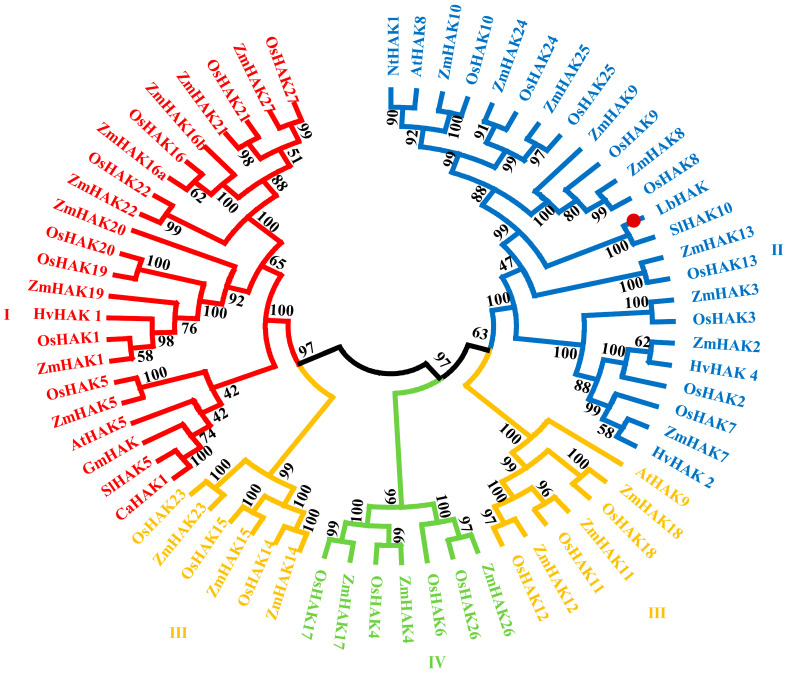
The phylogenetic tree of HAK/KUP/KT family of plant potassium transporters.

The complete cDNA sequence of LbHAK, which encodes a potassium transporter from the HAK/KUP/KT family in *L. barbarum*, was deposited in GenBank (accession NO. MZ416922.1). The *LbHAK* gene featured a 2004 bp ORF, potentially encoding 668 amino acids, and was predicted to have a molecular weight of 97.35 kDa (Table 1). The predicted isoelectric point of LbHAK was estimated as 9.07, furthermore LbHAK protein was predicted to be in the plasma membrane (Table 1). The deduced protein of LbHAK had ten transmembrane helices (Figure S2A). The outcome of multiple sequence alignment and phylogenetic tree analysis suggested that LbHAK is an orthologue of SlHAK10 and belongs to the cluster II of the HAK/KUP/KT family (Figure 1 and Figure S2B).

**Figure 2:**
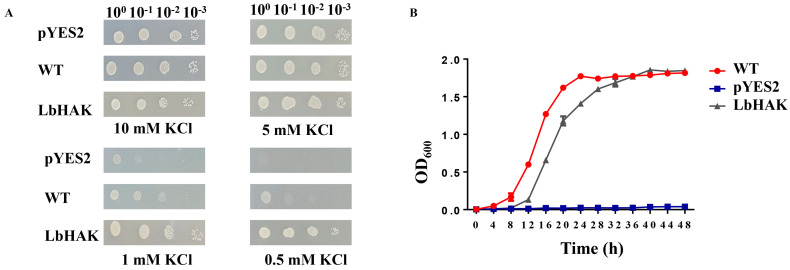
Heterogeneous expression of *LbHAK* in yeast CY162 strain to compensate the K acquisition. (**A**) Growth assay for CY162 with empty vector (pYES2) and expressing *LbHAK*, and corresponding wild-type strain BY4741 (WT) on AP medium contained 0.5, 1, 5, and 10 mM KCl. (**B**) Growth kinetic analysis of CY162 with empty vector (pYES2) and expressing *LbHAK*, and corresponding wild-type strain BY4741 (WT) cultured in liquid AP medium with 0.5 mM KCl. Data indicate as mean ± SD.

All the tested yeasts grew well on the AP medium at 10 mM and 5 mM KCl concentrations (Figure 2A). The potassium-uptake-defective yeast mutant CY162 with pYES2 showed growth inhibition at 1 mM KCl concentration and stopped growth at 0.5 mM KCl concentration (Figure 2A). Yeast mutant CY162 with LbHAK-pYES2 resembled the growth of wild-type yeast (BY4741) with pYES2 at 1 mM and 0.5 mM KCl concentrations (Figure 2A). Growth curve analysis showed that the growth rate of CY162 with LbHAK-pYES2 and wild-type yeast with pYES2 was significantly higher than that of the mutant yeast CY162 with pYES2 at 0.5 mM KCl concentration (Figure 2B). These data suggest the LbHAK can function as potassium transporter.

**Figure 3:**
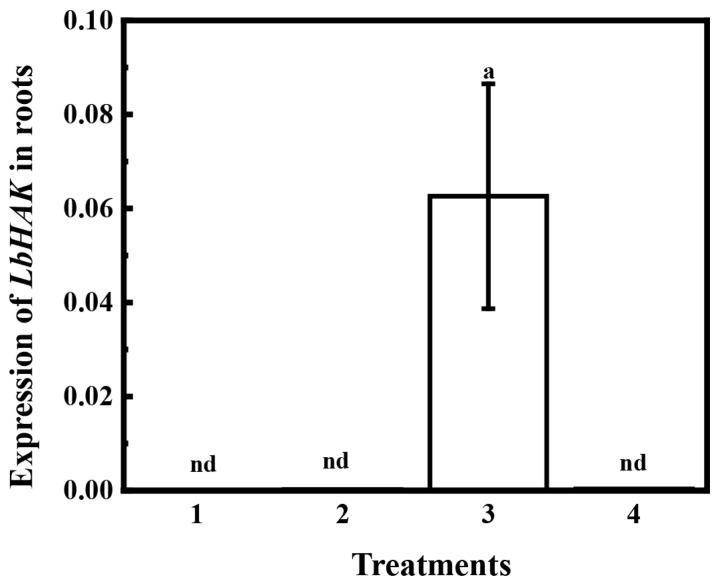
Expression of *LbHAK* in *L. barbarum* roots in the split-roots system. 1 and 2 represent two split roots from one plant that were not treated with AM inoculation, 3 and 4 represents two split roots from one plant that one part was inoculated with *R. irregularis* or without *R. irregularis*, respectively (see detail in Figure S1, nd represents not detected).

To characterize the expression patterns of *LbHAK* upon AM treatments in roots of *L. barbarum*, the split-root experiment was conducted (Figure S1). The *LbHAK* was only expressed in the AM-fungal-colonized root part of the AM treatment in the split-root experiment (Figure 3). No expression of *LbHAK* was detected in the non-colonized root part of the AM and NM treatments (Figure 3). These data suggest that the expression of *LbHAK* was specifically induced by AM in *L. barbarum* roots.

### 3.2. Overexpression of LbHAK Increased Growth and AM Colonization of Tobacco

**Figure 4:**
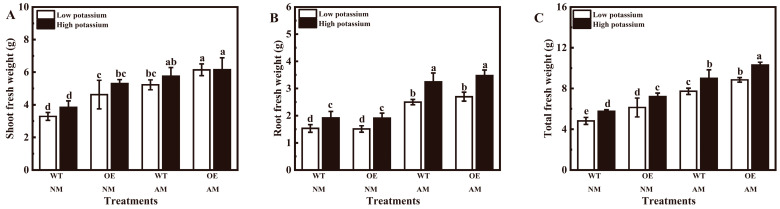
Effects of overexpression of *LbHAK* ((**A**), shoot; (**B**), root; (**C**), total) on tobacco growth. Low potassium: 0.2 mM kg^−1^ substrate; High potassium: 2 mM kg^−1^ substrate; WT, wild-type tobacco plants; OE, tobacco plants overexpressing *LbHAK*; NM, non-mycorrhizal; AM, inoculated with *R. irregularis*; bars indicate Mean ± SD (*n* = 4). Different letters above the bars indicate significant difference at *p* < 0.05 (Duncan test).

A total of 8 different transgenic tobacco lines overexpressing *LbHAK* were obtained (Figure S3). Inoculation of *R. irregularis* significantly improved the fresh weight of shoots, roots, and plants in WT and OE tobacco at 0.2 mM and 2 mM potassium conditions in the pot experiment (Table S3 and Figure 4A–C). The roots and total fresh weight of all tobacco were significantly increased under 2 mM potassium (Table S3 and Figure 4B,C). The overexpression of *LbHAK* led to a notable increase in the shoot and root fresh weight of non-mycorrhizal tobacco under two potassium conditions and in mycorrhizal tobacco under 0.2 mM potassium condition (Figure 4A,B). Additionally, it enhanced the total fresh weight of all tobacco plants under two potassium conditions (Figure 4C).

**Figure 5:**
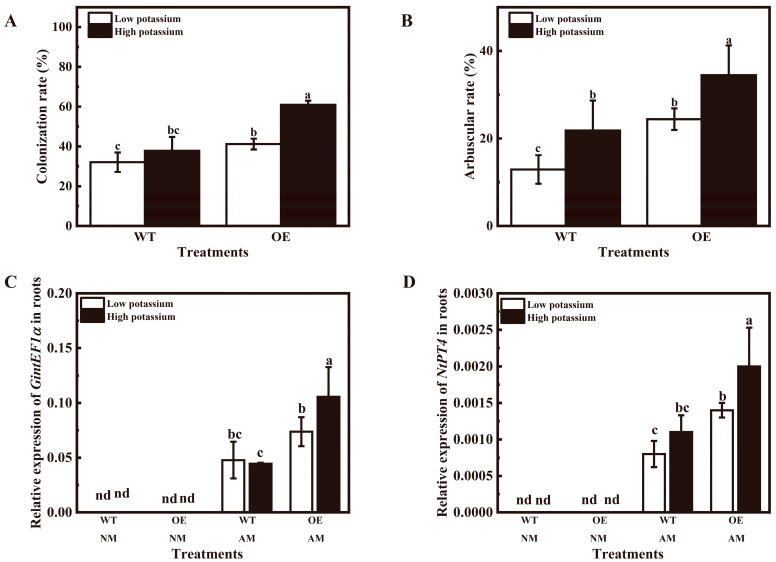
Evaluation of AM colonization efficiency in tobacco plants overexpressing *LbHAK*. (**A**) AM colonization rate; (**B**) arbuscular rate; *GintEF1α* (**C**) and *NtPT4* (**D**) relative expression in mycorrhizal tobacco plants. Low potassium: 0.2 mM kg^−1^ substrate; High potassium: 2 mM kg^−1^ substrate; WT, wild-type tobacco plants; OE, tobacco plants overexpressing *LbHAK*; NM, non-mycorrhizal; AM, inoculated with *R. irregularis*; nd, not detected. Bars indicate Mean ± SD (*n* = 4). Different letters above the bars indicate significant difference at *p* < 0.05 (Duncan test).

In addition to growth, OE tobacco plants showed an obviously increased mycorrhizal colonization rate and arbuscular rate at two potassium conditions in the pot experiment (Figure 5A,B). Application of 2 mM potassium also obviously increased the arbuscular rate in WT and OE tobacco (Figure 5B). The expression of *GintEF1α* and *NtPT4* were only expressed in mycorrhizal tobacco (Figure 5C,D). Overexpression of *LbHAK* significantly up-regulated *GintEF1α* expression of mycorrhizal tobacco at 2 mM potassium condition and mycorrhizal tobacco *NtPT4* expression at two potassium conditions (Figure 5C,D). Application of 2 mM potassium significantly enhanced the expression of *GintEF1*α and *NtPT4* in mycorrhizal OE tobacco plants (Figure 5C,D).

### 3.3. Overexpression of LbHAK Increased Tobacco Potassium and Phosphorus Uptake

**Figure 6:**
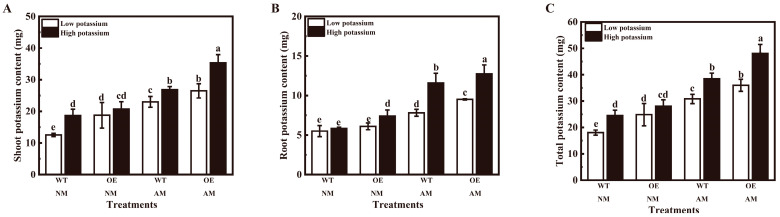
Overexpression of *LbHAK* improved potassium contents ((**A**), shoot; (**B**), root; (**C**), total) in tobacco plants. Low potassium: 0.2 mM kg^−1^ substrate; High potassium: 2 mM kg^−1^ substrate; WT, wild-type tobacco plants; OE, tobacco plants overexpressing *LbHAK*; NM, non-mycorrhizal; AM, inoculated with *R. irregularis*. Bars indicate Mean ± SD (*n* = 4). Different letters above the bars indicate significant difference at *p* < 0.05 (Duncan test).

Inoculation of *R. irregularis* significantly increased all tobacco shoot, root, and total potassium contents at two potassium conditions (Figure 6A–C). Application of 2 mM potassium significantly increased non-mycorrhizal WT tobacco shoot and total potassium contents and non-mycorrhizal OE tobacco root potassium content (Figure 6A–C). *LbHAK* overexpression significantly enhanced the non-mycorrhizal tobacco shoot and total potassium contents at 0.2 mM potassium condition and root potassium content at 2 mM potassium condition (Figure 6A–C).

**Figure 7:**
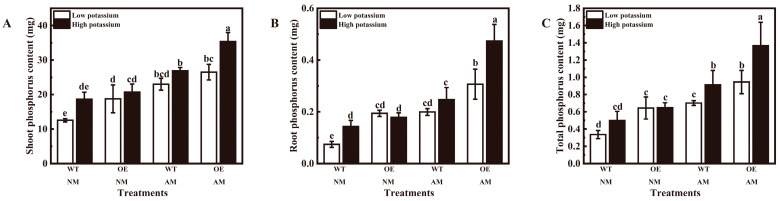
Overexpression of *LbHAK* improved phosphorus contents ((**A**), shoot; (**B**), root; (**C**), total)) in tobacco plants. Low potassium: 0.2 mM kg^−1^ substrate; High potassium: 2 mM kg^−1^ substrate; WT, wild-type tobacco plants; OE, tobacco plants overexpressing *LbHAK*; NM, non-mycorrhizal; AM, inoculated with *R. irregularis*. Bars indicate Mean ± SD (*n* = 4). Different letters above the bars indicate significant difference at *p* < 0.05 (Duncan test).

Additionally, inoculation of *R. irregularis* significantly increased the phosphorus contents in all tobacco shoots, roots, and whole plants irrespective of potassium conditions (Table S3 and Figure 7A–C). The mycorrhizal OE tobacco shoot, root, and total phosphorus contents were significantly increased under 2 mM potassium, as well as non-mycorrhizal WT tobacco root phosphorus content and mycorrhizal WT tobacco total phosphorus content (Table S3 and Figure 7A–C). *LbHAK* overexpression significantly increased the non-mycorrhizal tobacco shoot, root, and total phosphorus contents at 0.2 mM potassium condition; mycorrhizal tobacco shoot phosphorus content at 2 mM potassium condition; and root and total phosphorus contents at two potassium conditions (Table S3 and Figure 7A–C).

### 3.4. Overexpression of LbHAK Increased Rir-AQP1 and Rir-AQP2 Expression

**Figure 8:**
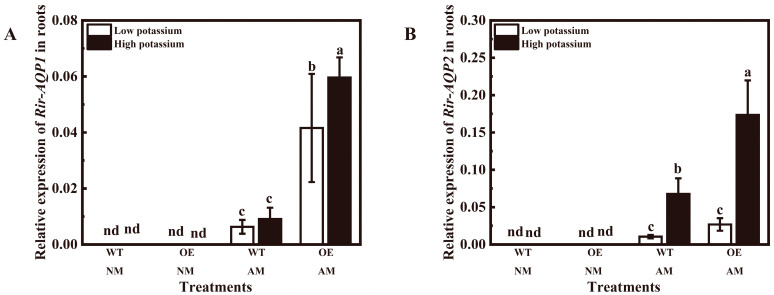
Expression levels of *Rir-AQP1* (**A**), and *Rir-AQP2* (**B**) in wild-type and *LbHAK*-overexpressing tobacco with or without AM treatments. Low potassium: 0.2 mM kg^−1^ substrate; High potassium: 2 mM kg^−1^ substrate; WT, wild-type tobacco plants; OE, tobacco plants overexpressing *LbHAK*; NM, non-mycorrhizal; AM, inoculated with *R. irregularis*; nd, not detected. Bars indicate Mean ± SD (*n* = 4). Different letters above the bars indicate significant difference at *p* < 0.05 (Duncan test).

The expression levels of two marker genes (*Rir-AQP1* and *Rir-AQP2*) were only detected in tobacco plants with mycorrhizal roots (Figure 8A,B). Overexpression of *LbHAK* significantly stimulated the expression levels of *Rir-AQP1* irrespective of potassium conditions and the expression of *Rir-AQP2* at 2 mM potassium condition (Table S3 and Figure 8A,B). Application of 2 mM potassium significantly increased the expression of *Rir-AQP1* in OE tobacco and the expression of *Rir-AQP2* in WT and OE tobacco (Table S3 and Figure 8A,B). These data suggest that overexpression of *LbHAK* may stimulate the expressions of *Rir-AQP1* and *Rir-AQP2*, thus improving the water transport capacities of host plants.

### 3.5. Correlation Analysis

**Figure 9:**
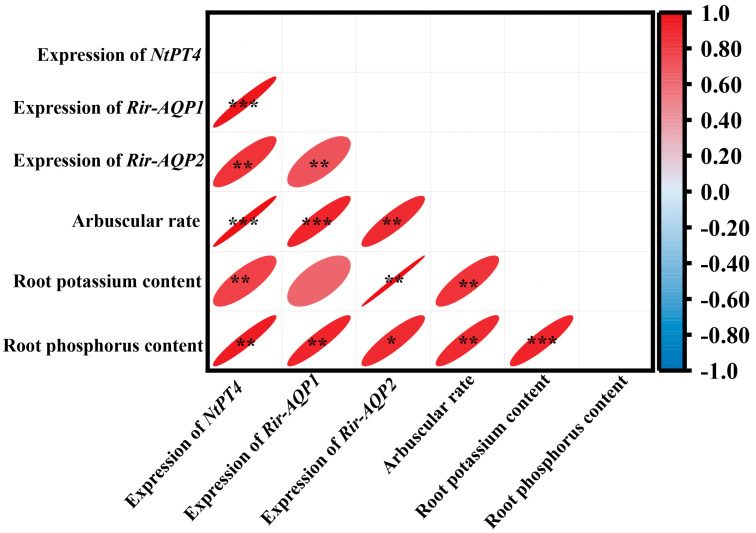
Correlation analysis of gene expression, root nutrient contents, and colonization rate in tobacco roots and *R. irregularis*. *, *p* < 0.05; **, *p* < 0.01; ***, *p* < 0.001.

To unravel the possible relationships between the detected parameters, correlation analysis was conducted (Figure 9). The mRNA abundance of the *NtPT4* gene was significantly positively correlated with mRNA levels of *Rir-AQP1* and *Rir-AQP2*, arbuscular rate, as well as the potassium and phosphorus contents in roots (Figure 9). Root potassium content was also closely positively correlated with the mRNA levels of two genes (*Rir-AQP1* and *Rir-AQP2*), arbuscular rate, and root phosphorus content (Figure 9).

## 4. Discussion

The plants colonized by AM fungi can not only directly uptake water and nutrients through plant roots, but also indirectly through AM fungal hyphae, which is known as the mycorrhizal pathway [4]. The mycorrhizal pathway is crucial for the absorption of Pi, and several AM-induced Pi transporters in plants have been identified [5,6]. Comparatively, the effects of AM symbiosis on the accumulation of potassium in plants received far less attention. Although there were reports that AM fungi improve plant potassium content, especially under potassium-limited condition [29,32], the direct evidence of the mycorrhizal pathway for potassium uptake was not discovered until the AM fungi specifically induced plant potassium transporter gene (*SlHAK10* in tomato) was functionally analyzed [30].

*L. barbarum*, which belongs to the Solanaceae family like tomato, tobacco, etc., could be colonized by AM fungi and nutritionally promoted by AM symbiosis [6,12,32,42]. A putative orthologue gene of *SlHAK10* in *L. barbarum* (designated as *LbHAK*) was isolated based on an independent de novo transcriptomic analysis (unpublished). The multiple sequence alignment and phylogenetic tree analysis indicated that *LbHAK* belongs to AM-induced cluster II of the HAK/KUP/KT family and has high similarity with *SlHAK10* (Figure 1 and Figure S2B). The heterologous yeast system certified the potassium transport capacity of LbHAK (Figure 2), and the expression of *LbHAK* was restricted within the AM-fungi-colonized root part (Figure 3). These results implied that *LbHAK* functions as a potassium transporter and may participate in the mycorrhizal pathway in *L. barbarum* as *SlHAK10* in tomato.

To further characterize the function of *LbHAK*, *LbHAK* was transformed into tobacco with the constitutive CaMV35S promoter. Despite the addition of potassium, *LbHAK* OE tobacco plants exhibited obvious growth promotion compared with WT tobacco plants (Figure 4). The result resembled our previous results when the potassium channel gene LbKAT3 [12] and ammonium transport gene *LbAMT3-1* [10] were overexpressed in tobacco. The CaMV35S promoter-driven nutrition uptake genes from *L. barbarum* perform functions in tobacco [43]. However, the overexpression of *SlHAK10* only promoted tomato growth with the inoculation of AM fungi under potassium-limited condition [30]. The potassium concentration in growth substrates and potassium demand of different plants may be the reason for this difference [12,30]. When the AM fungi were taken into consideration, the growth of *LbHAK* OE tobacco plants was further promoted. On one hand, this was a commonly observed result of the AM symbiosis effect on plant growth [33,39]. On the other hand, this may be a hint that *LbHAK* is in the preferred location in the peri-arbuscular membrane as its orthologue *SlHAK10* and performs potassium transport function for AM symbiosis.

In AM symbiosis, most nutrient transport through the mycorrhizal pathway takes place in the plant root cells harboring arbuscules [3,44]. Limitation of nutrient transfer from AM fungi to host plants usually exhibits limited or even eliminated arbuscular function and abundance [6,45]. In this study, *LbHAK* OE tobacco plants exhibited a highly increased colonization rate and arbuscular rate, and the increment was more obvious when a high amount of potassium was available (Figure 5). The expression of *NtPT4*, which is the indicator for the viability of arbuscules and phosphate transport in tobacco [7], was simultaneously increased in *LbHAK* OE tobacco plants. Although the variation of potassium availability in substrate did not show obvious influence on root colonization in *Medicago truncatula*, the expression of AM-specific marker gene (*MtPT4*) was up-regulated at high potassium condition [46]. The up-regulation of AM-specific phosphate transporter gene (*LbPT4*) was also observed when the folia-applied potassium was transferred via mycelia network to *L. barbarum* that were grown under potassium-limited condition [33]. These alike results suggested that the AM symbiosis has a promoted performance for phosphate transport under high potassium available condition [12].

More than promoted colonization, *LbHAK* OE tobacco also exhibited promoted potassium and phosphorus uptake, and the promotion was especially obvious when tobacco was colonized by AM fungi (Figure 6 and Figure 7). This was another piece of evidence for the up-regulated expression of *NtPT4* and the above-discussed point that AM symbiosis promotes plant phosphorus transport when more potassium is available. The simultaneously promoted potassium and phosphorus on one hand may be due to the co-distribution and a linked ratio by the nature of AM fungi (*R. irregularis*) [47,48]. On the other hand, the role of potassium as one of the major counter-ions of polyphosphate [49], which is the form of phosphate transfer along the hyphae of AM fungi to host plants, may explain the simultaneous potassium and phosphorus promotion.

Based on the model proposed by Kikuchi et al. (2016) [50], the transfer of polyphosphate along AM fungal hyphae required water flow that was facilitated by aquaporin in AM fungi. The expression of AM fungal aquaporin genes (*Rir-AQP1* and *Rir-AQP2*) was up-regulated in the *LbHAK* OE tobacco, especially under high potassium condition (Figure 8). Coincidentally, the expression of *NtPT4*, *Rir-AQP1*, *Rir-AQP2*, and the potassium and phosphorus contents in roots showed positive correlations (Figure 9). These results not only supported the model that the transport of phosphate via AM symbiosis requires the participation of AM fungal aquaporin, but also suggested a co-transport of phosphate and potassium in AM fungi [33,47,48]. Moreover, the arbuscular abundance, the expression of *NtPT4*, and the content of phosphorus and potassium in the mycorrhizal *LbHAK* OE tobacco plants were always higher than those in the mycorrhizal WT tobacco plants. Considering the mycorrhizal tobacco plants were under similar phosphate nutrient condition, the promoted function of AM symbiosis in the *LbHAK* OE tobacco suggested that there may be a regulatory mechanism between potassium and phosphorus in tobacco for the regulation of AM fungi [29].

## 5. Conclusions

In conclusion, *LbHAK* belongs to cluster II of the HAK/KUP/KT family. The expression of *LbHAK* is restricted within plant root parts that are colonized by AM fungi, and LbHAK may preferentially locate in the peri-arbuscular membrane. Compared with wild-type tobacco, *LbHAK*-overexpressing tobacco exhibited promoted growth, colonization rate, arbuscular abundance, and potassium and phosphorus content, especially when a high amount of potassium was available to the mycorrhizal plants. LbHAK may be involved in the mycorrhizal pathway for the potassium transport that accompanies phosphate transport. Future studies focused on the regulatory mechanism of plants on AM symbiosis between potassium and phosphorus are needed.

## Figures and Tables

**Table 1:** Bioinformation analysis of *LbHAK* in *L. barbarum*.

Gene Name	GenBank Accession NO.	ORF Length (bp)	Protein Length (bp)	Predicted Molecular Weight (kDa)	Isoelectric Point	Predicted Subcellular Location
*LbHAK*	MZ416922.1	2004	668	97.35	9.07	Plasma membrane

## Data Availability

The raw data supporting the conclusions of this article will be made available by the authors on request.

## Supplementary Materials

The following supporting information can be downloaded at: https://www.mdpi.com/article/10.3390/plants13091244/s1, Figure S1: Schematic diagram of the split-roots system; Figure S2: Bioinformation analysis of *LbHAK*; Figure S3: Detection of *LbHAK* expression in transgenic tobacco; Table S1: Primers used for clone and qRT-PCR; Table S2: Information for yeast strains and plasmids [34,35,51,52]; Table S3: ANOVA analysis of the detected parameters in this study.
