# Supplementary material for: Arbuscular Mycorrhizal Fungi Improve Lycium barbarum Potassium Uptake by Activating the Expression of LbHAK
Source: Plants (Basel). 2024 Apr 30;13(9):1244. doi: 10.3390/plants13091244 (PMC11085931; doi:10.3390/plants13091244)
Supplement: Supplementary file 1 [file plants-13-01244-s001.zip › plants-2973355-supplementary-figures.pdf]

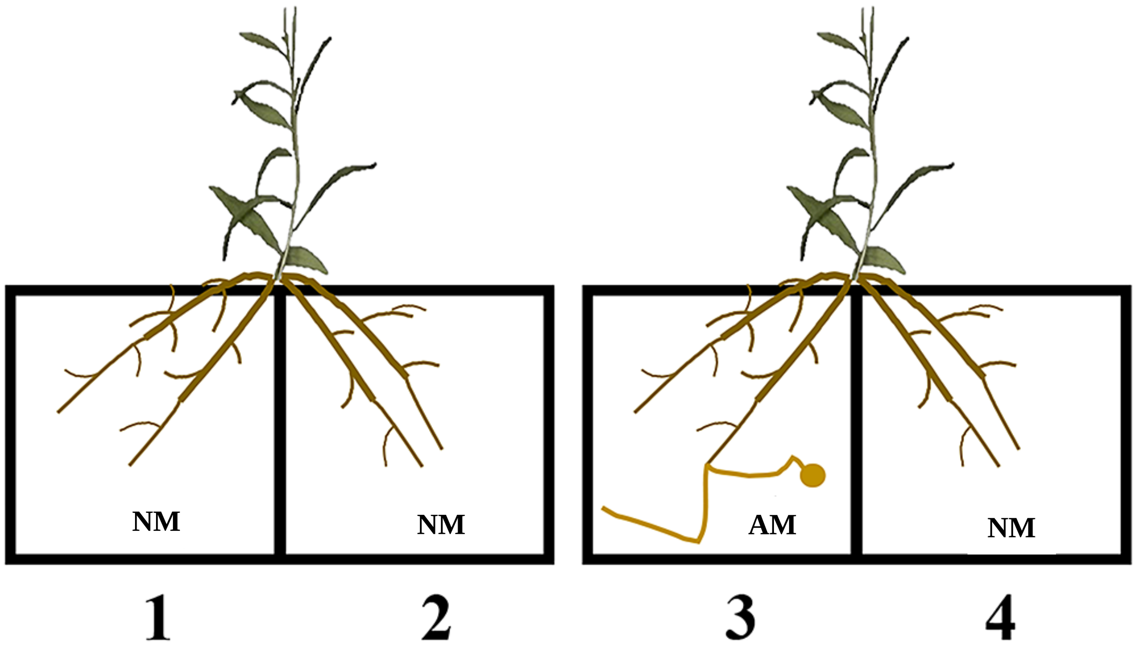


**Figure S1. Schematic diagram of the split-roots system.**
AM represents roots with AM inoculation, NM represents roots without AM inoculation, and the number at the bottom of the root compartment represents the four type roots.

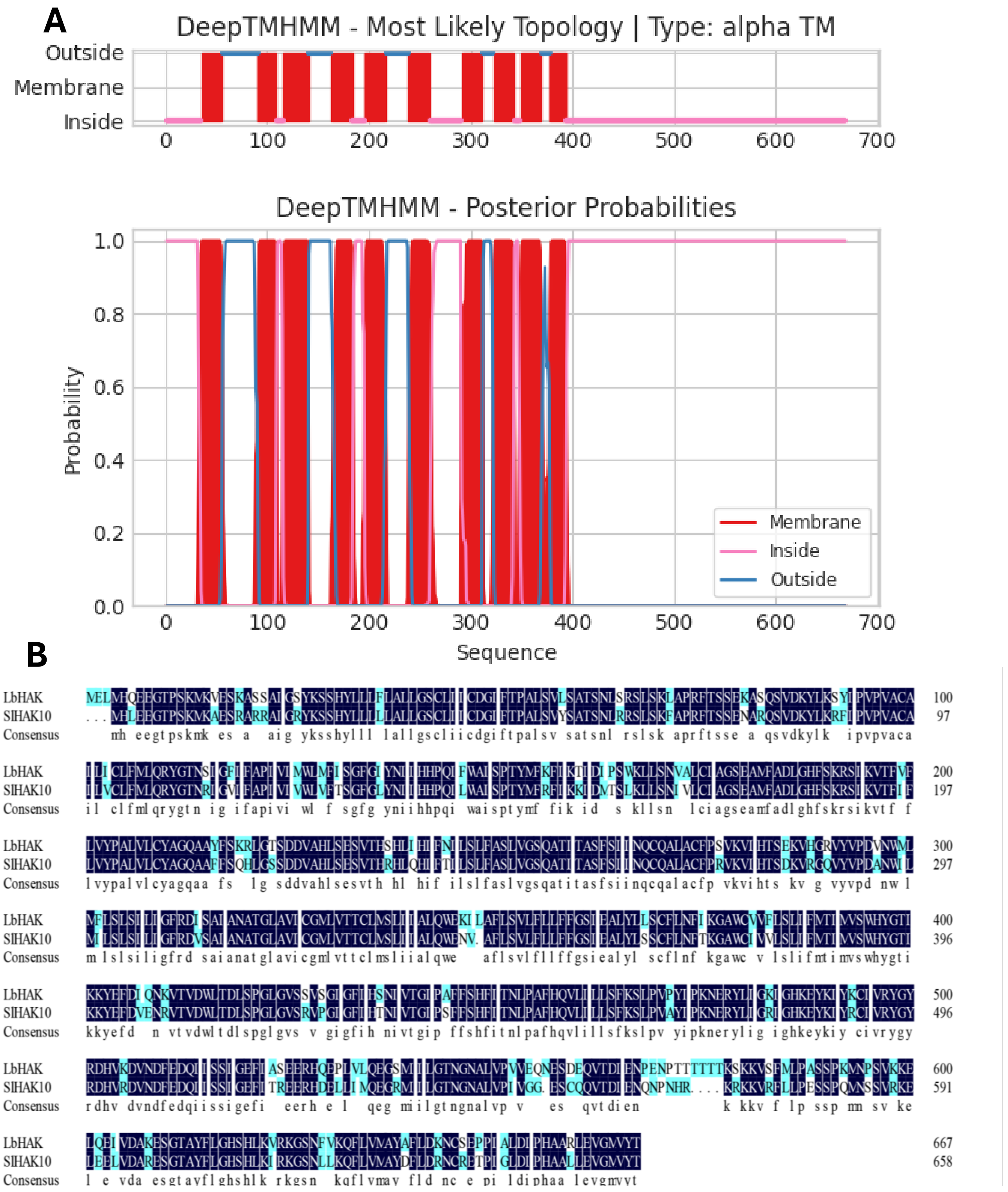


**Figure S2. Bioinformation analysis of *LbHAK*.** The putative transmembrane domains of LbHAK (A), and multiple alignment of the deduced amino acid sequences of *LbHAK* in *L. barbarum* with *SlHAK10* in tomato (B).

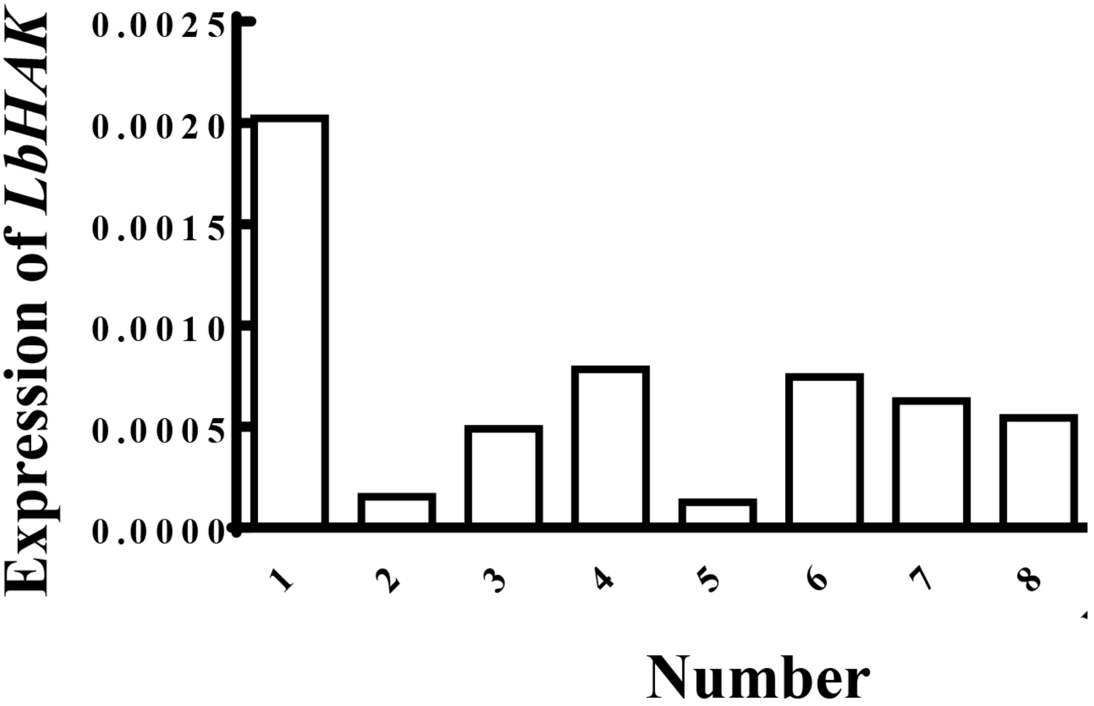


**Figure S3. Detection of *LbHAK* expression in different transgenic tobacco plants.**
The number represents different transgenic tobacco lines.
