# Supplementary material for: Arbuscular Mycorrhizal Fungi Improve Lycium barbarum Potassium Uptake by Activating the Expression of LbHAK
Source: Plants (Basel). 2024 Apr 30;13(9):1244. doi: 10.3390/plants13091244 (PMC11085931; doi:10.3390/plants13091244)
Supplement: Supplementary file 1 [file plants-13-01244-s001.zip › plants-2973355-supplementary-tables.pdf]

Table S1 Primers used for clone and quantitative real-time PCR (qRT-PCR)

Gene name	Primer names	Primer sequences (5'-3')	Used for	Annealing temperature (℃)
LbHAK	LbHAK-3outer	GTTGATGCAAGGGAAAGTGGCACTGCCT	3' RACE	55
	LbHAK-3inward	GTTGATGCAAGGGAAAGTGGCACTGCCT	3' RACE	55
	LbHAK-5outer	AAGGGCAAGAAGAGCGATTGGAAGATA	5' RACE	55
	LbHAK-5inward	CGTTCATTGTCAAAATTGGCACCCCGA	5' RACE	55
	LbHAK-f	CGTTTAGTCAATCCTCTCAGC	Full length PCR	55
	LbHAK-r	TCCCAGTCGTCGCAGAGTTAGAA	Full length PCR	55
LbActin	Q-LbActin-f	TCTACGAGGGTTACGCTTTG	qRT-PCR	55
	Q-LbActin-r	TCCCGTTCAGCAGTGGTT	qRT-PCR	55
LbHAK	Q-LbHAK-f	ACAAGGTGACTGTGGACTGGCT	qRT-PCR	55
	Q-LbHAK-r	TGGGAATGTATGGCACGGGC	qRT-PCR	55
NtPT4	Q-NtPT4-f	CGCTAGCAAAGCCCAACACAT	qRT-PCR	55
	Q- NtPT4-r	CGCTAGCAAAGCCCAACACAT	qRT-PCR	55
NtEF1α	Q-NtEF1α-f	TATGATTACTGGTACCTCCC	qRT-PCR	55
	Q-NtEF1α-r	ACCTAGCCTTGGAATACTTG	qRT-PCR	55
Rir-AQP1	Q-Rir-AQP1-f	CTTGCTATTCCATTCAGTTTCGG	qRT-PCR	55
	Q-Rir-AQP1-r	TTTTTTTTACTTTTTTGGGGTCC	qRT-PCR	55
Rir-AQP2	Q-Rir-AQP2-f	AGAGTCAGGAGGAGGAACAAGAG	qRT-PCR	55
	Q-Rir-AQP2-r	CTAACTGCAATACCCAAAGCGAG	qRT-PCR	55
GintEFα	Q-GintEFα-f	AATCAGTTGAAATGCACCACGAAC	qRT-PCR	55
	Q-GintEFα-f	CGACGAATTTCTTTGACTGATACGTT	qRT-PCR	55

Table S2 Information for yeast strains and plasmids.

Name	Description	Reference
CY162	*MATα ura3-52 his4-15 trk1Δ trk2Δ1::pCK64*	[1]
BY4741	Wild-type for CY162 strain	[2]
pYES2	Yeast gene expression plasmid	[3]
pROKII	Plant gene overexpressing plasmid	[4]

Reference:

1. Anderson, J.A.; Huprikar, S.S.; Kochian, L.V.; Lucas, W.J.; Gaber, R.F. Functional expression of a probable *Arabidopsis thaliana* potassium channel in *Saccharomyces cerevisiae*. Proceedings of the National Academy of Sciences 1992, 89, 3736-3740.

2. Winston, F.; Dollard, C.; Ricupero-Hovasse, S.L. Construction of a set of convenient *Saccharomyces cerevisiae* strains that are isogenic to S288C. Yeast 1995, 11, 53-55.

3. Reddy, A.; Maley, F. Studies on identifying the catalytic role of Glu-204 in the active site of yeast invertase. Journal of Biological Chemistry 1996, 271, 13953-13957, doi:10.1074/jbc.271.24.13953.

4. Liu, L.; Qu, J.; Wang, C.; Liu, M.; Zhang, C.; Zhang, X.; Guo, C.; Wu, C.; Yang, G.; Huang, J.; et al. An efficient genetic transformation system mediated by *Rhizobium rhizogenes* in fruit trees based on the transgenic hairy root to shoot conversion. Plant Biotechnology Journal 2024, doi:https://doi.org/10.1111/pbi.14328.

Table S3 ANONA analysis of the detected parameters in this study.

	Shoot fresh weight	Root fresh weight	Total Fresh weight	Shoot potassium content	Root potassium content	Total potassium content	Shoot phosphorus content	Root phosphorus content	Root phosphorus content
P_{AMF}	***	***	***	***	***	***	***	***	***
P_K	*	***	***	***	***	***	**	***	***
P_{Line}	***	ns	***	***	***	***	**	***	***
$P_{AMF} \times P_K$	ns	*	ns	ns	***	*	ns	**	*
$P_{AMF} \times P_{Line}$	ns	ns	ns	ns	ns	ns	ns	**	ns
$P_K \times P_{Line}$	ns	ns	ns	ns	ns	ns	ns	ns	ns
$P_{AMF} \times P_K \times P_{Line}$	ns	ns	ns	**	ns	*	ns	**	ns

	Colonization rate	Arbuscular rate	Relative expression of *GintEF1α* in roots	Relative expression of *NtPT4* in roots	Relative expression of *Rir-AQP1* in roots	Relative expression of *Rir-AQP2* in roots
P_{Line}	***	**	**	**	***	***
P_K	***	**	ns	ns	ns	***
$P_{Line} \times P_K$	*	ns	ns	ns	ns	**

Note: * P < 0.05; ** P < 0.01; *** P < 0.001; ns, no significant.
